# Supplementary material for: A predominant enhancer co-amplified with the SOX2 oncogene is necessary and sufficient for its expression in squamous cancer
Source: Nat Commun. 2021 Dec 8;12:7139. doi: 10.1038/s41467-021-27055-4 (PMC8654995; doi:10.1038/s41467-021-27055-4)
Supplement: Supplementary file 1 — Supplementary Information [file 41467_2021_27055_MOESM1_ESM.pdf]

A predominant enhancer co-amplified with the *SOX2* oncogene is necessary and sufficient for its expression in squamous cancer

Liu et al.

Supplementary Information

The Supplementary Information contains 10 Supplementary Figures (including figure legends) and 2 Supplementary Tables.

Supplementary Figure 1

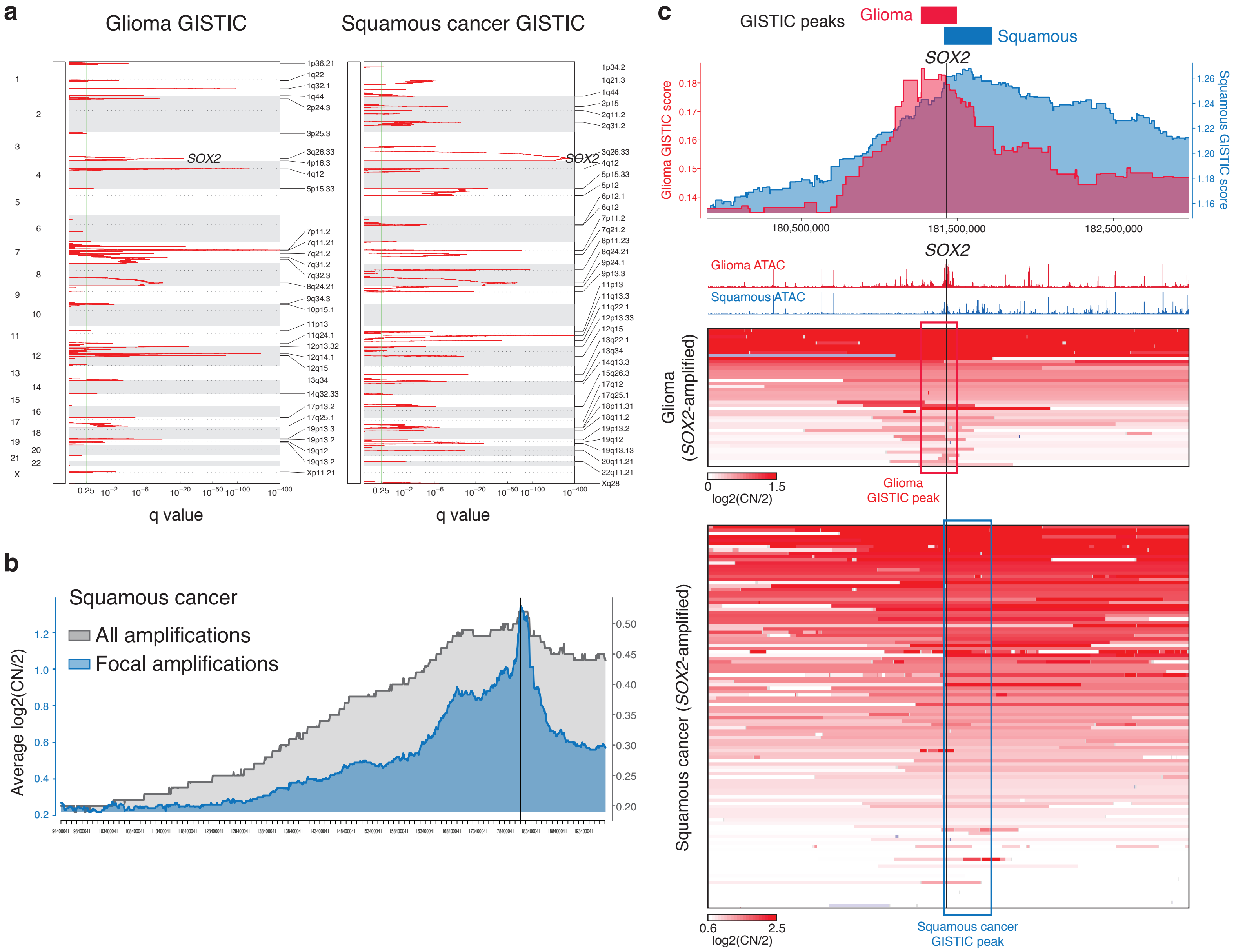

Supplementary Figure 1:

a. GISTIC results from TCGA glioma and squamous cancer samples

b. Profiles of the averaged copy number score (log2(copy number/2)) for copy number alterations from all squamous cancer samples or focal amplifications (<10 Mb) from samples that are associated with *SOX2* amplifications (log2(copy number/2) > 0.1).

c. Upper: distribution of GISTIC score in a ~5Mb windown centered at the *SOX2* gene. Middle: averaged ATAC-seq signal for TCGA glioma and squamous cancer samples. Bottom: copy number profiles of focal amplifications (<10 Mb) from samples that are associated with *SOX2* amplifications (log2(copy number/2) > 0.1).

Supplementary Figure 2

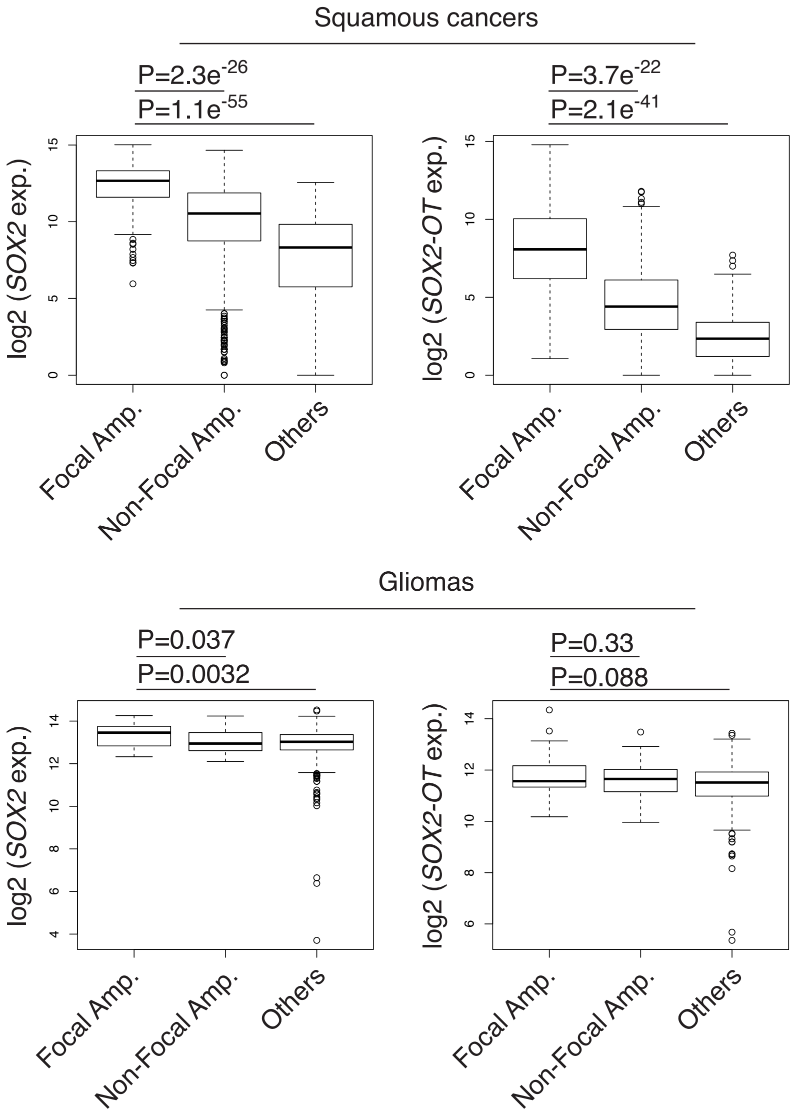

Supplementary Figure 2:
Expression levels of *SOX2* and *SOX2-OT* in TCGA samples with both RNA-seq and copy number data available. Upper: squamous cancers with *SOX2* focal amplifications (n=122 biologically independent samples), with *SOX2* non-focal amplifications (n=998 biologically independent samples), or without *SOX2* amplifications (n=258 biologically independent samples). Bottom: gliomas with *SOX2* focal amplifications (n=21), with *SOX2* non-focal amplifications (n=71), or without *SOX2* amplifications (n=563). Boxplots: middle bar, median; lower and upper box limits, the first and third quartiles, respectively; whiskers, the first quartile minus 1.5 times the interquartile range (IQR) and the third quartile plus 1.5 times the IQR, respectively. P values are derived from two-sided t-tests.

Supplementary Figure 3

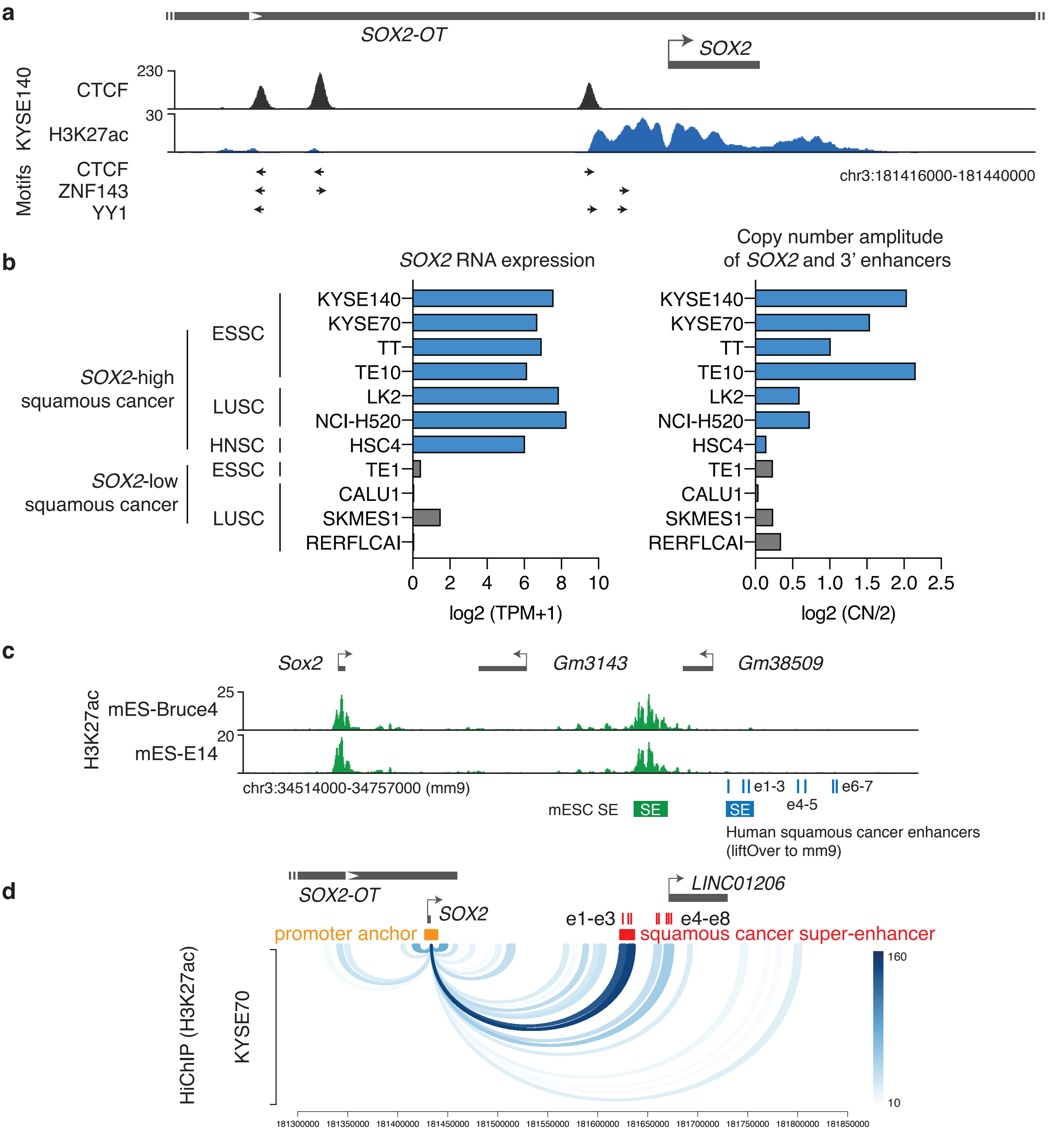

Supplementary Figure 3:

a. The CTCF ChIP-seq profile surrounding the *SOX2* promoter region. Underneath presented are motifs and their orientations of chromatin looping factors CTCF, ZNF143, and YY1.

b. Expression level (CCLE RNA-seq: log2(TPM+1)) and copy number score (CCLE log2(copy number/2)) of the *SOX2* region in squamous cancer cell lines.

c. The squamous cancer-specific enhancers and super-enhancer of *SOX2* (converted to mm9 genome) is distinct from the Sox2 super-enhancer reported for mouse ESC cells. Note: the enhancer e8 listed in Figure 2B has no corresponding region in the mouse mm9 genome (based on the liftOver tool).

d. H3K27ac HiChIP loops that are connected to the *SOX2* promoter in the squamous cancer cell line KYSE70. HiChIP anchors are defined by the union of H3K27ac peaks identified from the *SOX2*-high squamous cancer cell lines listed in Figure 2B. The color intensity corresponds to the number of PETs supporting each of the loops.

Source data are provided as a Source Data file.

Supplementary Figure 4

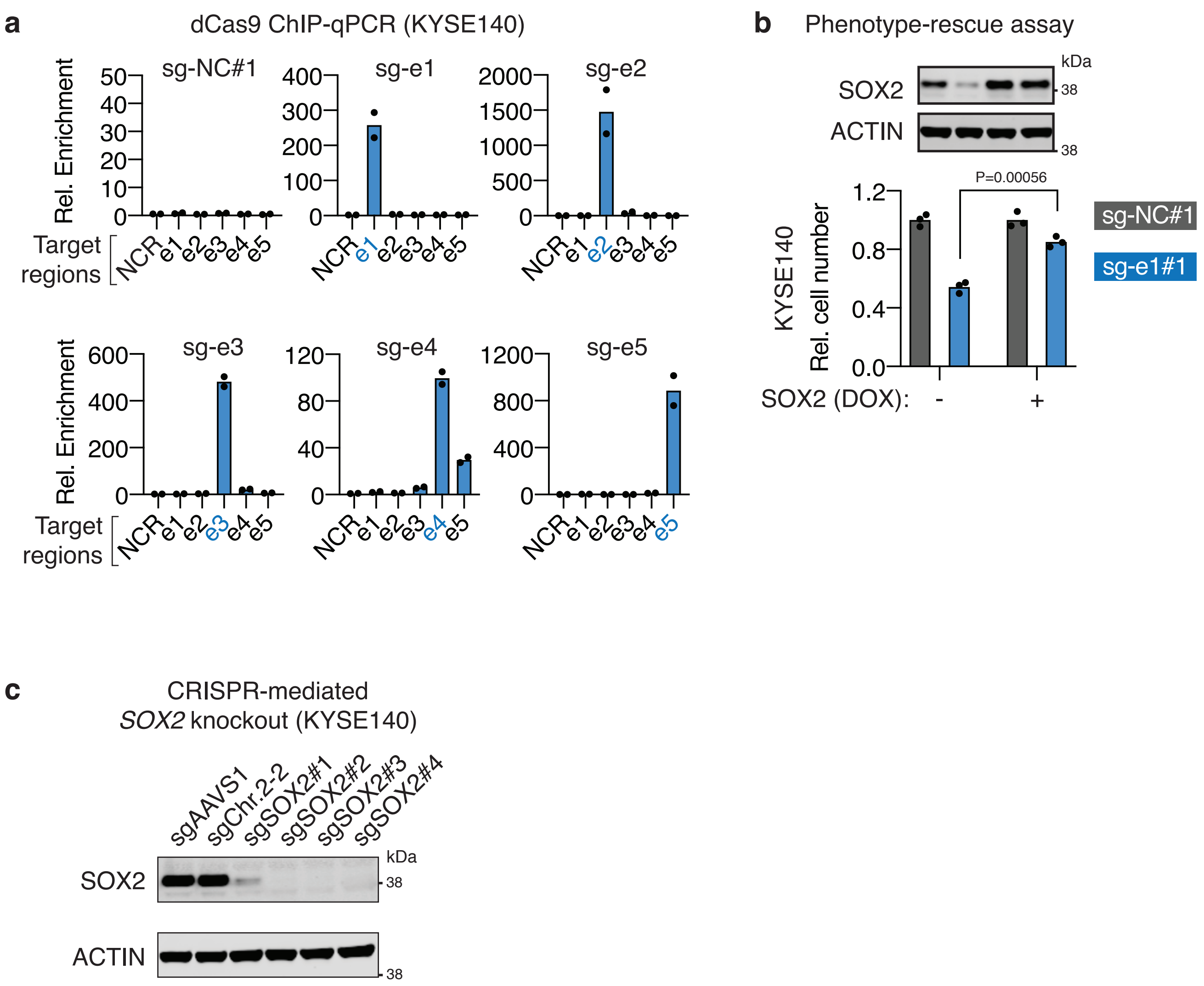

Supplementary Figure 4:

a. Cas9 ChIP-qPCR results showing enrichment of the dCas9-MeCP2-KRAB complex at each of the enhancers that are targeted by sgRNAs. For each region, ChIP-qPCR signal is normalized to sonicated genomic DNA. NCR: negative control region. n=2 biologically independent experiments.

b. Doxycycline-induced SOX2 expression in KYSE140 cells rescues the proliferation-inhibitory phenotype caused by e1 repression (six days post seeding the same number of cells with and without e1 repression). n=3 biologically independent experiments. P values are derived from a two-tailed t-test. The immunoblotting experiment was repeated once independently with similar results.

c. Immunoblots of SOX2 and ACTIN in KYSE140 cells with and without SOX2 knockouts (three days post selection). KYSE140 cells with all the conditions, except sgSOX2#1, were processed with RNA-seq to identify SOX2-regulated genes. The immunoblotting experiment was repeated once independently with similar results.

Source data are provided as a Source Data file.

Supplementary Figure 5

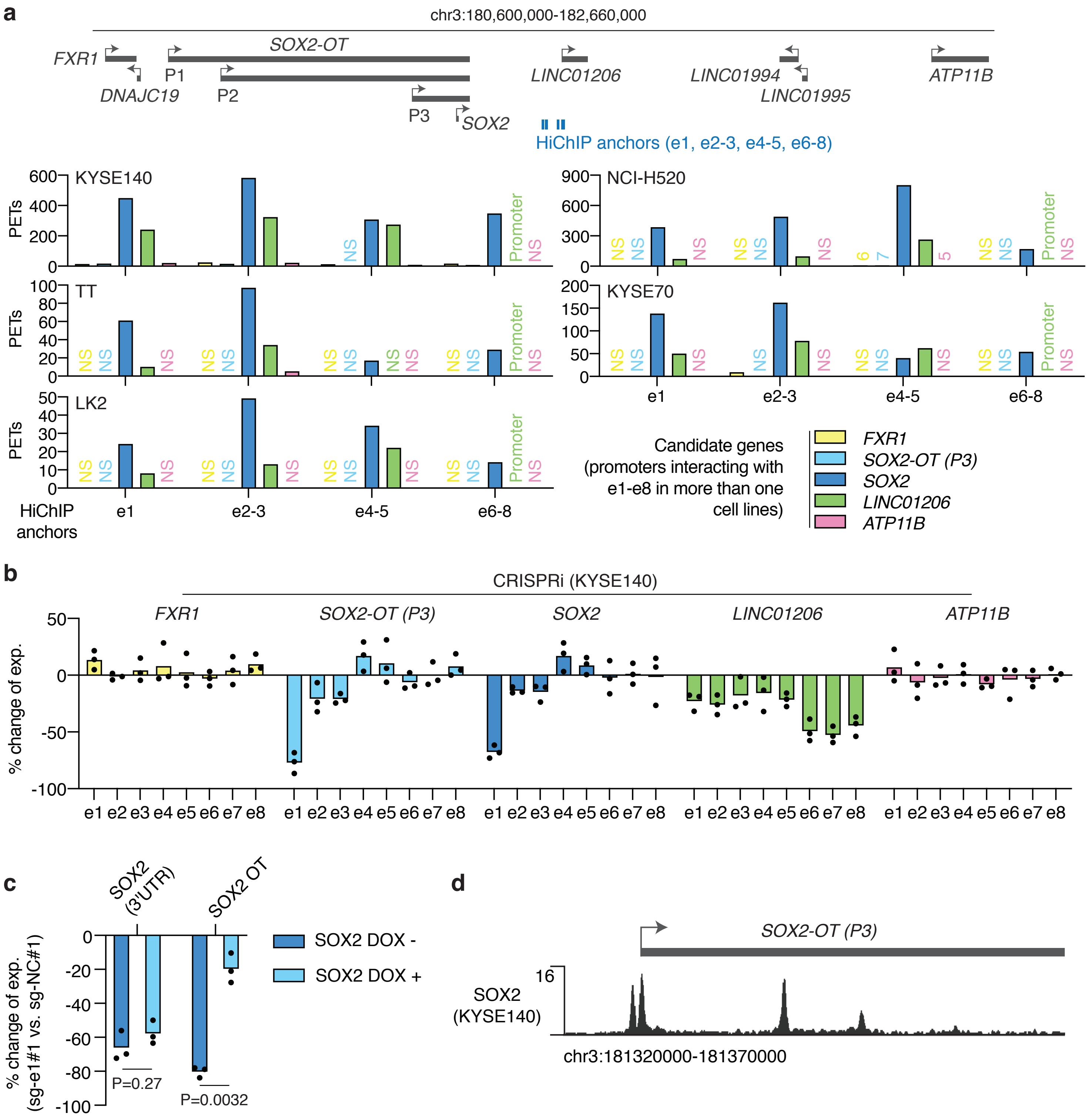

Supplementary Figure 5:

a. Number of PETs that connecting promoter regions of the candidate genes and the four HiChIP enhancer anchors. Note: The *SOX2-OT* gene has three alternative promoters - only the P3 promoter is connected to the enhancer anchors in two of the cell lines. N.S.: no significant loops.

b. Expression change (%) of each of the candidate genes in KYSE140 cells after individual repression of e1-e8. Expression level is normalized to the negative control (sg-NC#1). n=3 biologically independent experiments.

c. Expression change (%) of the endogenous *SOX2* and *SOX2-OT* after e1 repression in KYSE140 cells with and without ectopic expression of *SOX2*. Primers targeting the *SOX2* 3'UTR region were used to distinguish the endogenous and ectopic *SOX2*. n=3 biologically independent experiments. P value is derived from two-sided t-test.

d. SOX2 ChIP-seq profile at the *SOX2-OT* (P3 promoter isoform) locus.

Source data are provided as a Source Data file.

Supplementary Figure 6

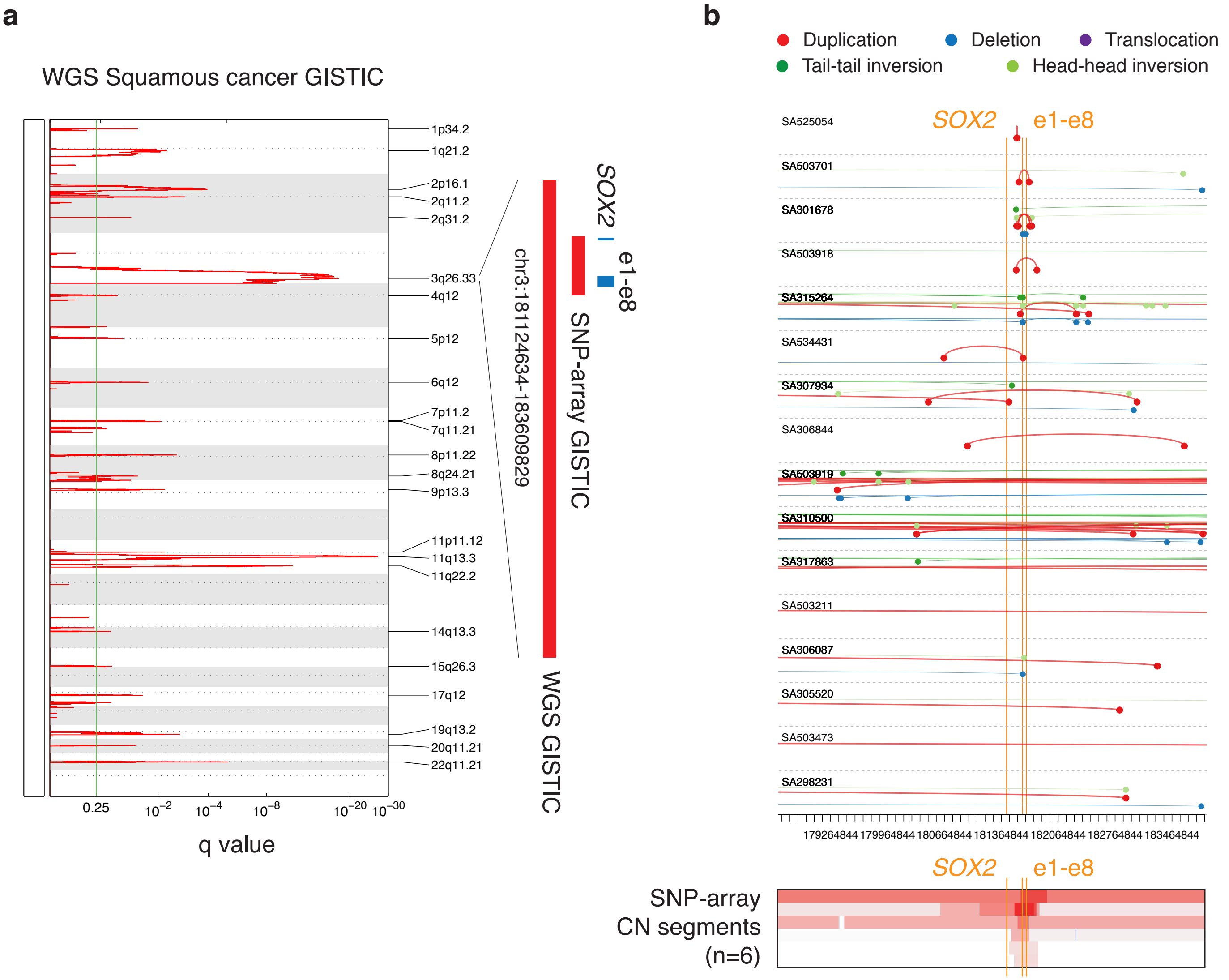

Supplementary Figure 6:
a. GISTIC result from squamous cancer WGS data.
b. Upper: Structural variants identified by WGS analysis at the *SOX2*-e1 locus in squamous cancers. Bottom: SNP-array-based copy number data showing squamous cancer samples that have amplifications of the enhancer region alone.

Supplementary Figure 7

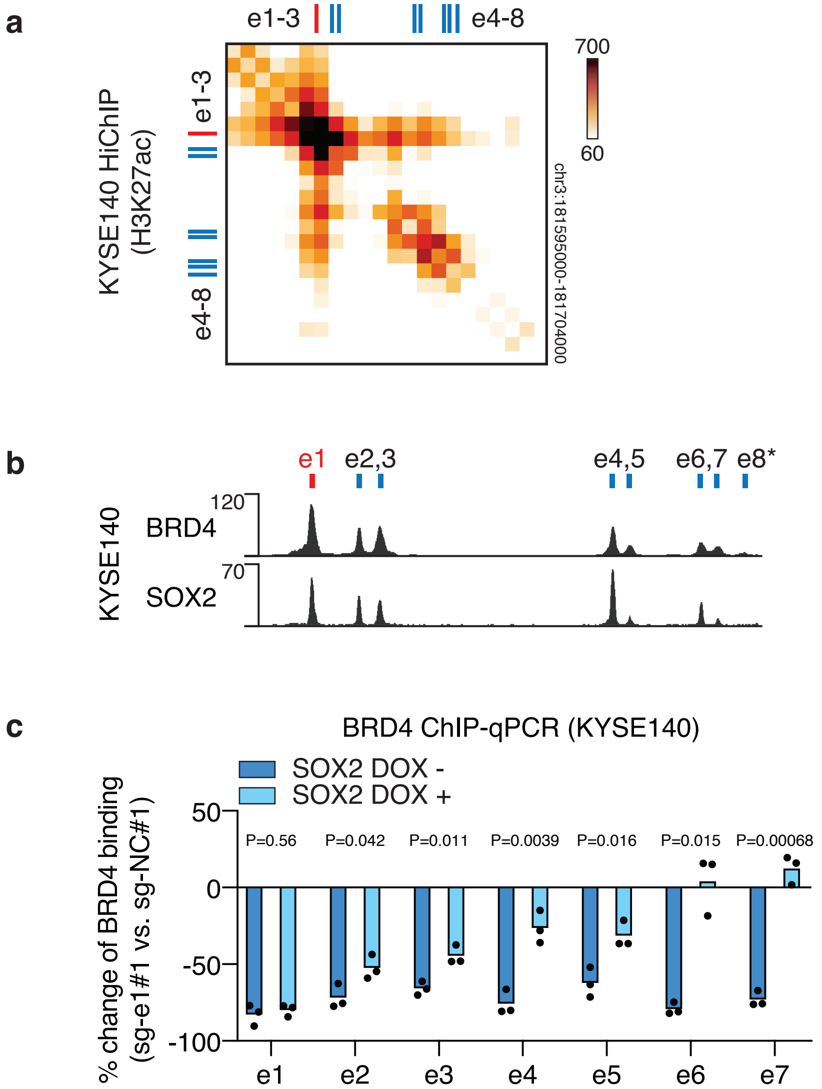

Supplementary Figure 7:

a. H3K27ac HiChIP contact heatmap showing the interaction between e1 and the other enhancers in the squamous cancer enhancer cluster in KYSE140 cells.

b. ChIP-seq of BRD4 and SOX2 signal at the e1-e8 locus. *: based on peak calling, e8 has no significant enrichment of BRD4 in KYSE140 cells. BRD4 ChIP-seq data was from KYSE140 sg-NC#1.

c. BRD4 ChIP-qPCR results showing the % change of BRD4 binding at e1-e7 after e1 repression in KYSE140 cells with and without Doxycycline-induced ectopic expression of *SOX2*. n=3 biologically independent experiments. P values are derived from two-sided t-tests.

Source data are provided as a Source Data file.

Supplementary Figure 8

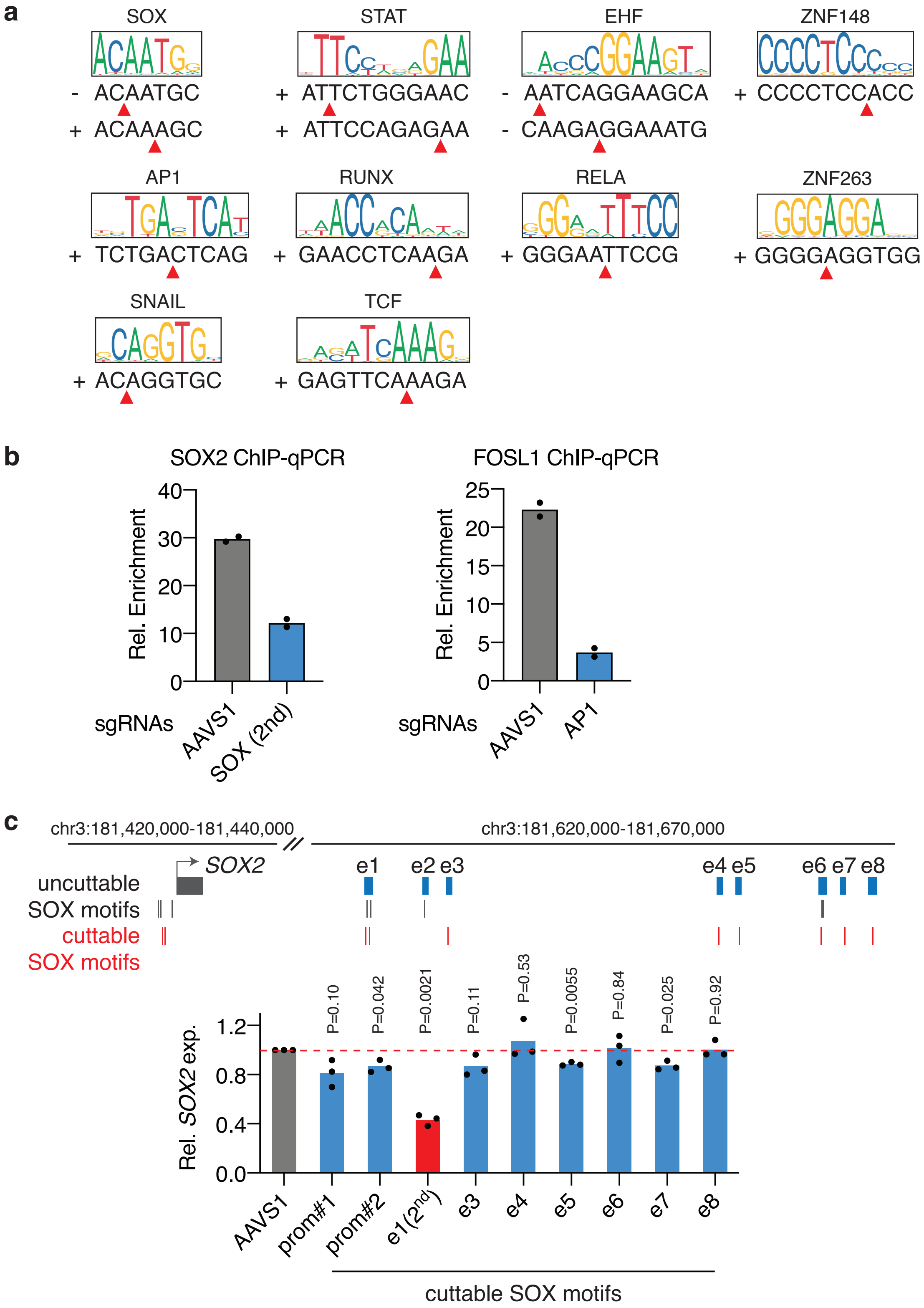

Supplementary Figure 8:
a. List of motifs that are disrupted by CRISPR cutting.
b. Left: SOX2 ChIP-qPCR at the e1 enhancer in KYSE140 cells with and without CRISPR-mediated disruption of the 2nd SOX2 motif found in e1. Right: FOSL1 ChIP-qPCR at the e1 enhancer in KYSE140 cells with and without CRISPR-mediated disruption of the AP-1 motif found in e1. ChIP enrichment was normalized to DNA concentration of each sample (measured by Qubit) and then to sonicated genomic input. n=2 biologically independent experiments.
c. Expression of *SOX2* in KYSE140 cells with cutting of SOX motifs in e2-e8 and *SOX2* promoter. The expression levels are normalized to the AAVS1 negative control. The 2nd SOX motif in e1 serves as a positive control for the experiment. n=3 biologically independent experiments. P values are derived from two-sided t-tests.
Source data are provided as a Source Data file.

Supplementary Figure 9

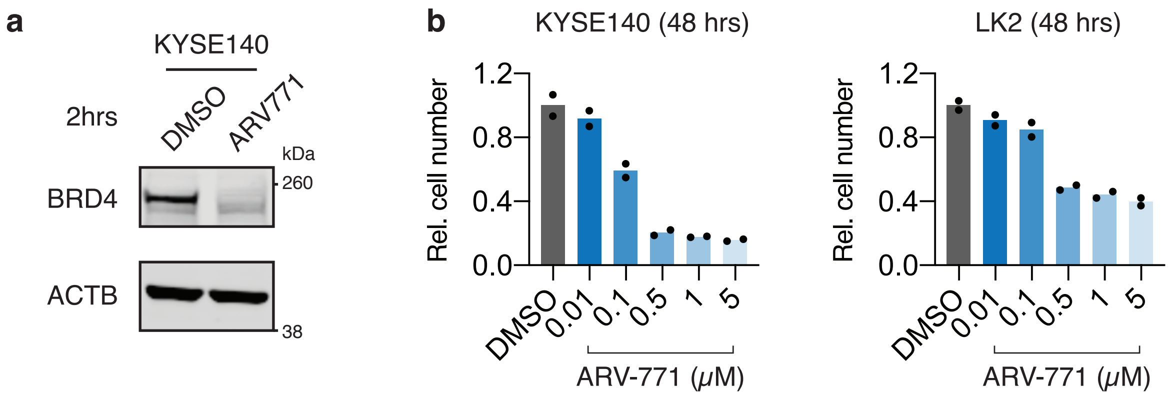

Supplementary Figure 9:
a. Immunoblots of BRD4 and ACTIN in KYSE140 cells treated with two hours of DMSO or 0.5 μM ARV-771. The immunoblotting experiment was repeated once independently with similar results.
b. Cell proliferation assays of KYSE140 and LK2 cells treated with 48 hours of DMSO or ARV-771. Cell numbers were normalized to the DMSO controls. n=2 biologically independent experiments.
Source data are provided as a Source Data file.

Supplementary Figure 10

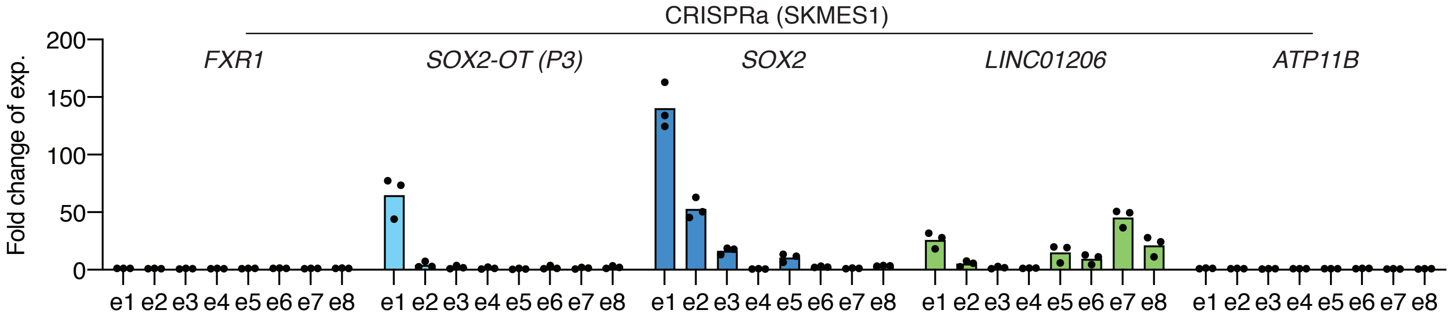

Supplementary Figure 10:
Expression fold change of *FXR1*, *SOX2-OT* (P3 promoter isoform), *SOX2*, *LINC01206*, and *ATP11B* in SKMES1 cells after CRISPR-mediated activation of e1-e8. The expression levels are normalized to the sg-NC#1 negative control. n=3 biologically independent experiments.
Source data are provided as a Source Data file.

Table S1: KEGG pathway analysis for e1-activated genes

Collection(s):	CP:KEGG
# overlaps shown:	20
# genesets in collections:	186
# genes in comparison (n):	1526
# genes in universe (N):	40071

Gene Set Name	# Genes in Gene Set (K)	Description	# Genes in Overlap (k)	k/K	p-value	FDR q-value
KEGG_AXON_GUIDANCE	129	Axon guidance	22	0.1705	3.76E-09	5.68E-07
KEGG_LYSOSOME	121	Lysosome	21	0.1736	6.11E-09	5.68E-07
KEGG_MAPK_SIGNALING_PATHWAY	267	MAPK signaling pathway	32	0.1199	1.20E-08	7.46E-07
KEGG_VALINE_LEUCINE_AND_ISOLEUCINE_DEGRADATION	44	Valine, leucine and isoleucine degradation	11	0.25	5.68E-07	2.53E-05
KEGG_REGULATION_OF_ACTIN_CYTOSKELETON	213	Regulation of actin cytoskeleton	25	0.1174	6.81E-07	2.53E-05
KEGG_PATHWAYS_IN_CANCER	325	Pathways in cancer	32	0.0985	1.11E-06	3.44E-05
KEGG_GLYCINE_SERINE_AND_THREONINE_METABOLISM	31	Glycine, serine and threonine metabolism	9	0.2903	1.55E-06	4.06E-05
KEGG_VASCULAR_SMOOTH_MUSCLE_CONTRACTION	115	Vascular smooth muscle contraction	17	0.1478	1.74E-06	4.06E-05
KEGG_BASAL_CELL_CARCINOMA	55	Basal cell carcinoma	11	0.2	6.03E-06	1.25E-04
KEGG_NEUROTROPHIN_SIGNALING_PATHWAY	126	Neurotrophin signaling pathway	16	0.127	2.50E-05	4.65E-04
KEGG_MELANOGENESIS	101	Melanogenesis	14	0.1386	2.95E-05	4.99E-04
KEGG_BUTANOATE_METABOLISM	34	Butanoate metabolism	8	0.2353	3.25E-05	5.04E-04
KEGG_GAP_JUNCTION	90	Gap junction	13	0.1444	3.62E-05	5.18E-04
KEGG_HEDGEHOG_SIGNALING_PATHWAY	56	Hedgehog signaling pathway	10	0.1786	4.49E-05	5.96E-04
KEGG_CALCIUM_SIGNALING_PATHWAY	178	Calcium signaling pathway	19	0.1067	5.39E-05	6.68E-04
KEGG_ENDOCYTOSIS	181	Endocytosis	19	0.105	6.77E-05	7.86E-04
KEGG_TGF_BETA_SIGNALING_PATHWAY	86	TGF-beta signaling pathway	12	0.1395	1.01E-04	1.10E-03
KEGG_ARRHYTHMOGENIC_RIGHT_VENTRICULAR_CARDIOMYOPATHY_ARVC	74	Arrhythmogenic right ventricular cardiomyopathy (ARVC)	11	0.1486	1.09E-04	1.12E-03
KEGG_CHEMOKINE_SIGNALING_PATHWAY	189	Chemokine signaling pathway	19	0.1005	1.21E-04	1.14E-03
KEGG_GNRH_SIGNALING_PATHWAY	101	GnRH signaling pathway	13	0.1287	1.22E-04	1.14E-03

Table S2: Primers used in the study

sgRNAs for CRISPRi and CRISPRa

Names	Sequences	BLAT regions (hg19)	Notes
sg_NC1_F	CACCGATCGTTTCCGCTTAACGGCG	N/A	Negative control
sg_NC1_R	AAACCGCCGTTAAGCGGAAACGATC		
sg_NC2_F	CACCGCTGAGTGAAAAATAAAAGTT	N/A	Negative control
sg_NC2_R	AAACAACTTTTATTTTTCACTCAGC		
sg_Prom_F	CACCGCCCCCTTTCATGCAAAACC	chr3:181429603-181429622	
sg_Prom_R	AAACGGTTTTGCATGAAAGGGGGC		
sg_e1_1_F	CACCGGCAAACCTTGAAACAAAGC	chr3:181625876-181625895	Also used for cutting the 2nd SOX motif at e1
sg_e1_1_R	AAACGCTTTGTTTCAAGGTTTGCC		
sg_e1_2_F	CACCGCTCAAGATGAACGTTCCCAC	chr3:181625972-181625991	
sg_e1_2_R	AAACGTGGGAACGTTCATCTTGAGC		
sg_e1_3_F	CACCGTCAGAAAACCTGCACCTGTG	chr3:181626079-181626098	Also used for cutting the SNAIL motif at e1
sg_e1_3_R	AAACCACAGGTGCAGGTTTTCTGAC		
sg_e2_F	CACCGATGTCTTCACATATGACTGG	chr3:181631224-181631243	
sg_e2_R	AAACCCAGTCATATGTGAAGACATC		
sg_e3_F	CACCGCTAATACCAACTCCAACTGC	chr3:181633517-181633536	
sg_e3_R	AAACGCAGTTGGAGTTGGTATTAGC		
sg_e4_F	CACCGTAAATCTTAGCGTTGGTAA	chr3:181659352-181659371	
sg_e4_R	AAACTTACCAACGCTAAGATTTAC		
sg_e5_F	CACCGCATGCTCTAAGACCCTAAGG	chr3:181661141-181661160	
sg_e5_R	AAACCCTTAGGGTCTTAGAGCATGC		
sg_e6_F	CACCGTCCTGACGTAATGAGATACT	chr3:181669171-181669190	
sg_e6_R	AAACAGTATCTCATTACGTCAGGAC		
sg_e7_F	CACCGTTATGTCCCATCCTTTCACA	chr3:181671025-181671044	
sg_e7_R	AAACTGTGAAAGGATGGGACATAAC		
sg_e8_F	CACCGGTACACCCAGACAGAGTGA	chr3:181674059-181674078	
sg_e8_R	AAACTCACTCTGTCTGGGTGTACC		

sgRNAs for CRISPR-mediated motif cutting within the e1 enhancer
sg_e1_1 was used to cut the 2nd SOX motif and sg_e1_3 was used to cut the SNAIL motif

Names	Sequences	BLAT regions (hg19)	Notes
sg_e1_1stSOX_F	CACCGCTTGTGTGATTTGCATTGTT	chr3:181625525-181625544	1st SOX motif
sg_e1_1stSOX_R	AAACAACAATGCAAATCACACAAGC		
sg_e1_1stSTAT_F	CACCGCTGCTAGAAGCCAGCATTCT	chr3:181625563-181625582	1st STAT motif
sg_e1_1stSTAT_R	AAACAGAATGCTGGCTTCTAGCAGC		
sg_e1_1stEHF_F	CACCGTTAAACAAAAGCAACAAATC	chr3:181625637-181625656	1st EHF motif
sg_e1_1stEHF_R	AAACGATTTGTTGCTTTTGTTTAAC		
sg_e1_2ndEHF_F	CACCGTTTAAGAGACATTTCCTCT	chr3:181625651-181625670	2nd EHF motif
sg_e1_2ndEHF_R	AAACAGAGGAAATGTCTCTTAAAC		
sg_e1_ZNF148_F	CACCGAATAAAAGGAACGTGGTGGA	chr3:181625851-181625870	ZNF148 motif
sg_e1_ZNF148_R	AAACTCCACCACGTTCCTTTTATTC		
sg_e1_AP1_F	CACCGGTGGTGGAGGTTCTGACTC	chr3:181625923-181625942	AP1 motif
sg_e1_AP1_R	AAACGAGTCAGAACCTCCACCACC		
sg_e1_RUNX_F	CACCGCTGTGGGAACGTTCATCTTG	chr3:181625974-181625993	RUNX motif
sg_e1_RUNX_R	AAACCAAGATGAACGTTCCCACAGC		
sg_e1_2ndSTAT_F	CACCGAGCAGGCAGCTTTGTTTCTC	chr3:181626001-181626020	2nd STAT motif
sg_e1_2ndSTAT_R	AAACGAGAAACAAAGCTGCCTGCTC		
sg_e1_RELA_F	CACCGAACATTCACCTTGGGAATTC	chr3:181626036-181626055	RELA motif
sg_e1_RELA_R	AAACGAATTCCCAAGGTGAATGTTC		
sg_e1_ZNF263_F	CACCGTTCCGGCAGCACCGGGGAGG	chr3:181626053-181626072	ZNF263 motif
sg_e1_ZNF263_R	AAACCCTCCCCGGTGCTGCCGGAAC		
sg_e1_TCF_F	CACCGGAGTTGCCAGAGTTCAAAG	chr3:181626110-181626129	TCF motif
sg_e1_TCF_R	AAACCTTTGAACTCTGGCAACTCC		

sgRNAs for CRISPR-mediated cutting of SOX motifs outside of e1

Names	Sequences	BLAT regions (hg19)	Notes
prom_SOX_1st_F	CACCGAATCTTTACGTCGGGACAAT	chr3:181428321-181428340	
prom_SOX_1st_R	AAACATTGTCCCGACGTAAAGATTC		
prom_SOX_2nd_F	CACCGGGCTTTGCGGCCACCACAA	chr3:181428571-181428590	
prom_SOX_2nd_R	AAACTTGTGGTGGCCGCAAAGCCC		
e3_SOX_F	CACCGTCCTGATACTTAGGGAACAA	chr3:181633327-181633346	
e3_SOX_R	AAACTTGTTCCCTAAGTATCAGGAC		
e4_SOX_F	CACCGTTCTGTTCTTTGCATACAAT	chr3:181659264-181659283	
e4_SOX_R	AAACATTGTATGCAAAGAACAGAAC		
e5_SOX_F	CACCGAAAGCTTCAAGCGAACAATG	chr3:181661244-181661263	
e5_SOX_R	AAACCATTGTTCGCTTGAAGCTTTC		
e6_SOX_F	CACCGTCACTATGCAGAAGAAACAA	chr3:181669056-181669075	
e6_SOX_R	AAACTTGTTTCTTCTGCATAGTGAC		
e7_SOX_F	CACCGACTGAAAAACTTAAACACAA	chr3:181671304-181671323	
e7_SOX_R	AAACTTGTGTTTAAGTTTTTCAGTC		
e8_SOX_F	CACCGTGGGAACATTCACAACAAA	chr3:181674027-181674046	
e8_SOX_R	AAACTTTGTTGTGAATGTTCCCAC		

sgRNA for CRISPR-mediated SOX2 knockout

Names	Sequences	BLAT regions (hg19)	Notes
sg_AAVS1_F	CACCGAGCCACATTAACCGGCCCT	chr19:55627180-55627199	Negative control
sg_AAVS1_R	AAACAGGGCCGGTTAATGTGGCTC		
sg_chr22_F	CACCGGTGTGCGTATGAAGCAGTG	chr2:151131560-151131579	Negative control
sg_chr22_R	AAACCACTGCTTCATACGCACACC		
sg_SOX2_1_F	CACCGTCGCCATGCTATTGCCGCCG	chr3:181430550-181430569	
sg_SOX2_1_R	AAACCGGCGGCAATAGCATGGCGAC		
sg_SOX2_2_F	CACCGAAAGTTTCCACTCGGCGCCC	chr3:181430373-181430392	
sg_SOX2_2_R	AAACGGGCGCCGAGTGGAAACTTTC		
sg_SOX2_3_F	CACCGCAAGCGCCTGGGCGCCGAG	chr3:181430363-181430382	
sg_SOX2_3_R	AAACCTCGGCGCCCAGGCGCTTGC		
sg_SOX2_4_F	CACCGCGGCAACCAGAAAAACAGCC	chr3:181430241-181430260	
sg_SOX2_4_R	AAACGGCTGTTTTTCTGGTTGCCGC		

RT-qPCR

Names	Sequences	Target genes	Notes
RT_ACTB_F	CAACCGCGAGAAGATGACC	ACTB	
RT_ACTB_R	AGCCTGGATAGCAACGTACA		
RT_HPRT1_F	GACCAGTCAACAGGGGACAT	HPRT1	
RT_HPRT1_R	CCTGACCAAGGAAAGCAAAG		
RT_GAPDH_F	GGAGCGAGATCCCTCCAAAAT	GAPDH	
RT_GAPDH_R	GGCTGTTGTCATACTTCTCATGG		
RT_SOX2_1_F	ACCAGCTCGCAGACCTACAT	SOX2 (exon)	
RT_SOX2_1_R	TGGAGTGGGAGGAAGAGGTA		
RT_SOX2_2_F	GGAGCTTTGCAGGAAGTTTG	SOX2 (3'UTR)	
RT_SOX2_2_R	GCAAGAAGCCTCTCCTTGAA		
RT_FXR1_F	GTGGCAAATCCTCCATCAGT	FXR1	
RT_FXR1_R	ACCGCCTACGACGGTTAGTA		
RT_ATP11B_F	TGGACTCCATGTGCTGTTTC	ATP11B	
RT_ATP11B_R	TCAAGATGCTCCTGTCGTTG		
RT_SOX2OT_F	TTTGAAAACTCCCCTTGCAC	SOX2OT (P3)	
RT_SOX2OT_R	GAAGAGGGACCAGCTGACTG		
RT_LINC01206_F	AGGCTTAGGCAATGGGAAGT	LINC01206	
RT_LINC01206_R	TGCCTTGCTTTCCTCTCACT		

ChIP-qPCR

Names	Sequences	Amplified regions (hg19)	Notes
ChIP_NCR_F	TGGGTGGTGTCATCTGGTAA	chr3:129250860-129250952	Negative control region
ChIP_NCR_R	GGATGGAATGGATCAGATGG		
ChIP_NCR2_F	GCTGCTTTAGTGGCTGTTCC	chr11:112964712-112964844	Negative control region
ChIP_NCR2_R	GCAGCTGCCGTCTTAACTGT		
ChIP_SOX2_prom_F	TGGTCGCTAGAAACCCATTT	chr3:181429668-181429764	
ChIP_SOX2_prom_R	TCTGCCTTGACAACTCCTGA		
ChIP_SOX2_e1_F	CCTCCACCACGTTCCTTTTA	chr3:181625849-181625947	
ChIP_SOX2_e1_R	AGCCTGAGTCAGAACCTCCA		
ChIP_SOX2_e2_F	CCTGACACCAGCAATGAATG	chr3:181631166-181631252	
ChIP_SOX2_e2_R	TCCTGGGACATGTCTTCACA		
ChIP_SOX2_e3_F	GAGCAACTTAGCAGCCATCC	chr3:181633496-181633595	
ChIP_SOX2_e3_R	CTGACCTCGTCCCAACTTTC		
ChIP_SOX2_e4_F	AGAGCCGTTACCAACGCTAA	chr3:181659345-181659414	
ChIP_SOX2_e4_R	GACGGCTGTTTGGAACATTT		
ChIP_SOX2_e5_F	CGAGGGATACCCCTTTAAGC	chr3:181661191-181661260	
ChIP_SOX2_e5_R	TGTTCGCTTGAAGCTTTCCT		
ChIP_SOX2_e6_F	GTGAGGGGTGGATTACCAGA	chr3:181669354-181669457	
ChIP_SOX2_e6_R	GGACTCCCAAAGTGCTGAGA		
ChIP_SOX2_e7_F	GCCGATCACAGCTACCTCAT	chr3:181671396-181671499	
ChIP_SOX2_e7_R	GCTGATGGTCGTTTGGATTT		

Uncropped immunoblots included in supplementary figures

Supplementary Fig. 4b

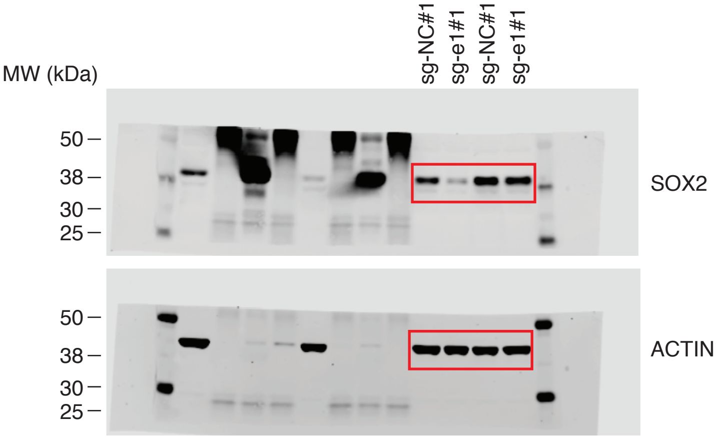

The above two images are from the same membrane that was scanned with two separate channels in LI-COR.

Supplementary Fig. 4c

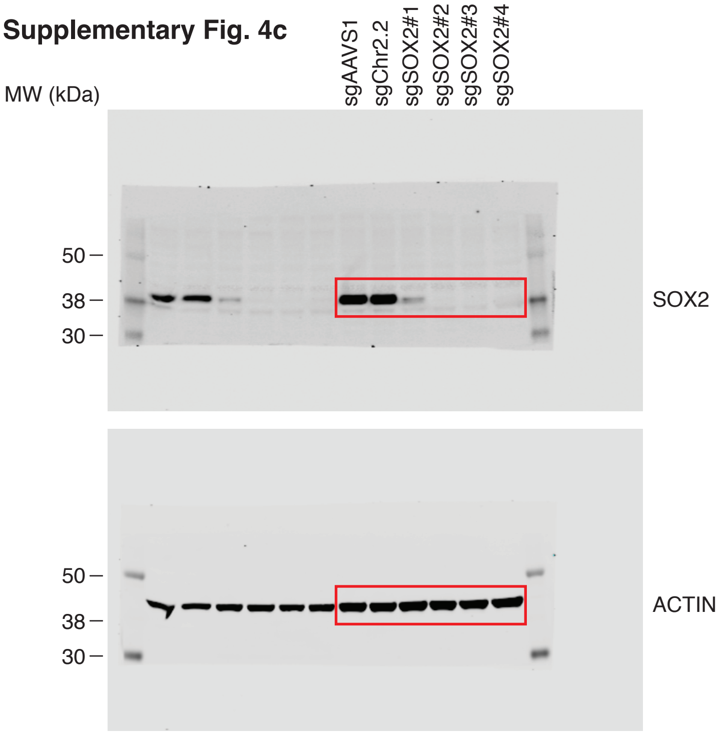

The above two images are from the same membrane that was scanned with two separate channels in LI-COR.

Supplementary Fig. 9a

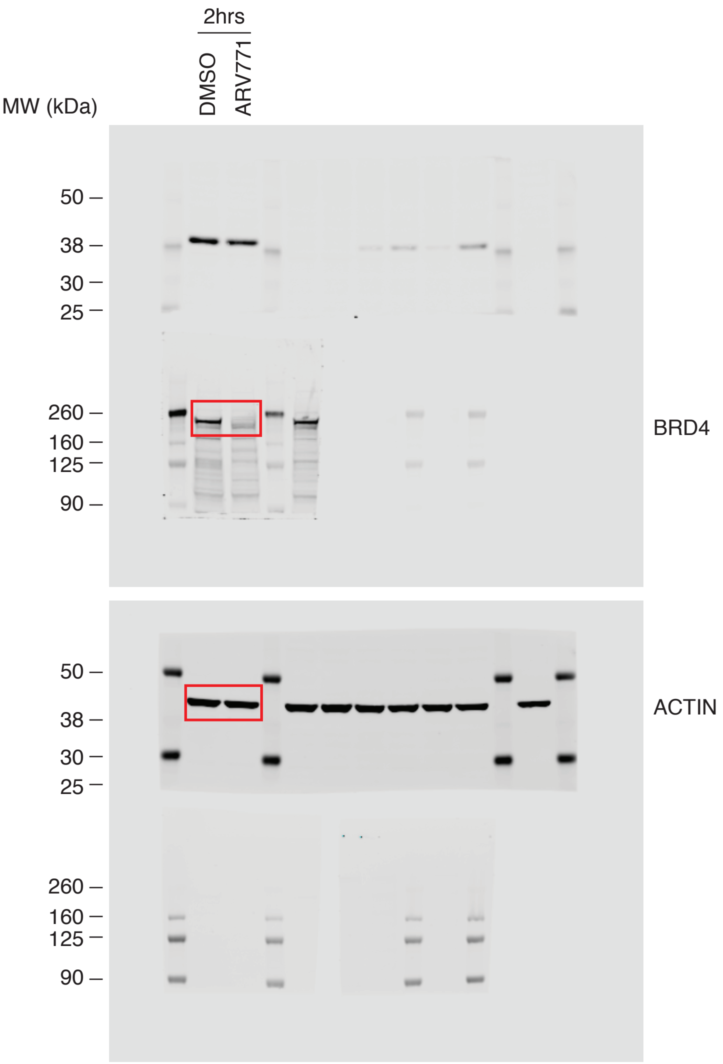

The above two images are from the same set of membranes that were scanned with two separate channels in LI-COR.
