# Supplementary material for: A predominant enhancer co-amplified with the SOX2 oncogene is necessary and sufficient for its expression in squamous cancer
Source: Nat Commun. 2021 Dec 8;12:7139. doi: 10.1038/s41467-021-27055-4 (PMC8654995; doi:10.1038/s41467-021-27055-4)
Supplement: Supplementary file 3 — Reporting Summary [file 41467_2021_27055_MOESM3_ESM.pdf]

nature research

Corresponding author(s): Xiaoyang Zhang
Swneke D. Bailey

Last updated by author(s): Oct 28, 2021

Reporting Summary

Nature Research wishes to improve the reproducibility of the work that we publish. This form provides structure for consistency and transparency in reporting. For further information on Nature Research policies, see our Editorial Policies and the Editorial Policy Checklist.

Statistics

For all statistical analyses, confirm that the following items are present in the figure legend, table legend, main text, or Methods section.

n/a	Confirmed	
☐	☒	The exact sample size (*n*) for each experimental group/condition, given as a discrete number and unit of measurement
☐	☒	A statement on whether measurements were taken from distinct samples or whether the same sample was measured repeatedly
☐	☒	The statistical test(s) used AND whether they are one- or two-sided<br>*Only common tests should be described solely by name; describe more complex techniques in the Methods section.*
☒	☐	A description of all covariates tested
☐	☒	A description of any assumptions or corrections, such as tests of normality and adjustment for multiple comparisons
☐	☒	A full description of the statistical parameters including central tendency (e.g. means) or other basic estimates (e.g. regression coefficient) AND variation (e.g. standard deviation) or associated estimates of uncertainty (e.g. confidence intervals)
☐	☒	For null hypothesis testing, the test statistic (e.g. *F*, *t*, *r*) with confidence intervals, effect sizes, degrees of freedom and *P* value noted<br>*Give P values as exact values whenever suitable.*
☒	☐	For Bayesian analysis, information on the choice of priors and Markov chain Monte Carlo settings
☒	☐	For hierarchical and complex designs, identification of the appropriate level for tests and full reporting of outcomes
☒	☐	Estimates of effect sizes (e.g. Cohen's *d*, Pearson's *r*), indicating how they were calculated

Our web collection on statistics for biologists contains articles on many of the points above.

Software and code

Policy information about availability of computer code

Data collection	No softwares were used for data collection
Data analysis	Softwares that were used for data analysis:<br>Bowtie2 (2-2.2.9) for ChIP-seq and RNA-seq reads alignment<br>HiC-Pro (2.11.1) for HiChIP reads alignment<br>samtools (1.12) for sorting and indexing alignments<br>MACS2 (2.2.6) for ChIP-seq peak calling<br>bedtools (2.28.0) for ChIP-seq peaks comparison<br>RSEM (1.3.0) for quantifying RNA-seq reads<br>edgeR (3.24.3) for differential gene expression analysis<br>hichipper (0.7.0) for HiChIP loop calling<br>Homer (v4.11) for super-enhancer identification<br>FIMO (5.0.5) for transcription factor motifs identification<br>deepTools (3.3.0) for ChIP-seq signal presentation

For manuscripts utilizing custom algorithms or software that are central to the research but not yet described in published literature, software must be made available to editors and reviewers. We strongly encourage code deposition in a community repository (e.g. GitHub). See the Nature Research guidelines for submitting code & software for further information.

Data

Policy information about availability of data

All manuscripts must include a data availability statement. This statement should provide the following information, where applicable:

- Accession codes, unique identifiers, or web links for publicly available datasets
- A list of figures that have associated raw data
- A description of any restrictions on data availability

TCGA publicly available copy number and ATAC-seq data was downloaded from NCI Genomic Data Commons data portal (copy number URL: https://gdc.cancer.gov/about-data/publications/pancanatlas; ATAC-seq URL: https://gdc.cancer.gov/about-data/publications/ATACseq-AWG). TCGA publicly available RNA-seq data was downloaded from Broad Institute GDAC data portal (URL: http://gdac.broadinstitute.org/). PCAWG publicly available whole-genome sequencing data was downloaded from PCAWG data portal (URL: http://gdac.broadinstitute.org/). The H3K27ac ChIP-seq publicly available data used in this study were downloaded from the Gene Expression Omnibus (GEO) series GSE137461 (URL: https://www.ncbi.nlm.nih.gov/geo/query/acc.cgi?acc=GSE137461, for LK2, NCI-H520, and CALU1 cells), GSE88976 (URL: https://www.ncbi.nlm.nih.gov/geo/query/acc.cgi?acc=GSE88976, for TT and TE10 cells), GSE16256 (URL: https://www.ncbi.nlm.nih.gov/geo/query/acc.cgi?acc=GSE16256, for hESC), and GSE31039 (URL: https://www.ncbi.nlm.nih.gov/geo/query/acc.cgi?acc=GSE31039, for mESC). The ChIP-seq, HiChIP, and RNA-seq data generated in this study have been deposited to GEO under the series GSE166234 (URL: https://www.ncbi.nlm.nih.gov/geo/query/acc.cgi?acc=GSE166234). Source data are provided with this paper. The remaining data are available within the Article, Supplementary Information or Source Data file.

Field-specific reporting

Please select the one below that is the best fit for your research. If you are not sure, read the appropriate sections before making your selection.

[x] Life sciences [ ] Behavioural & social sciences [ ] Ecological, evolutionary & environmental sciences

For a reference copy of the document with all sections, see nature.com/documents/nr-reporting-summary-flat.pdf

Life sciences study design

All studies must disclose on these points even when the disclosure is negative.

Sample size	Sample size is described in figure legends of the manuscript. We did not perform any computation to pre-determine sample sizes. We chose the sample sizes based on previous literature, common standards in the field, our own experience, and experimental feasibility. The results suggest that the chosen sample size is appropriate because of statistical significance or observed clear distinctions.
Data exclusions	No data was excluded
Replication	Two or three biological replicates were performed for molecular and cellular assays and 10 mice (per condition) were used for xenograft experiments. Replicates yielded similar results as shown in the figures.
Randomization	Randomization was performed at the beginning of xenograft experiments. No randomization was applied to the other experiments as they were based on in vitro cells grouped by distinct genetic perturbations or drug treatments.
Blinding	Blinding was not applied to the study as the grouping information was necessary for data acquisition and interpretation.

Reporting for specific materials, systems and methods

We require information from authors about some types of materials, experimental systems and methods used in many studies. Here, indicate whether each material, system or method listed is relevant to your study. If you are not sure if a list item applies to your research, read the appropriate section before selecting a response.

Materials & experimental systems

n/a	Involved in the study	
[ ]	[x]	Antibodies
[ ]	[x]	Eukaryotic cell lines
[x]	[ ]	Palaeontology and archaeology
[ ]	[x]	Animals and other organisms
[x]	[ ]	Human research participants
[x]	[ ]	Clinical data
[x]	[ ]	Dual use research of concern

Methods

n/a	Involved in the study	
[ ]	[x]	ChIP-seq
[x]	[ ]	Flow cytometry
[x]	[ ]	MRI-based neuroimaging

Antibodies

Antibodies used	Antibodies used for ChIP or HiChIP:<br>H3K27ac, Abcam, ab4729, rabbit polyclonal (amount: 4ug/ChIP or 7.5ug/HiChIP)

	H3K27ac, Active Motif, 39133, rabbit polyclonal (amount: 7.5ug/HiChIP)<br>BRD4, Bethyl, A301-985A100, rabbit polyclonal (amount: 4ug/ChIP)<br>SOX2, R&D Systems, AF2018, goat polyclonal (amount: 4ug/ChIP)<br>CTCF, Cell Signaling, 2899, rabbit polyclonal (amount: 10ul/ChIP)<br>FOSL1, Cell Signaling, 5281, rabbit monoclonal, clone D80B4 (amount: 10ul/ChIP)<br>Cas9, Diagenode, C15310258, rabbit polyclonal (amount: 4ug/ChIP)<br><br>Antibodies used for Immunoblotting:<br>SOX2, Cell Signaling, 3728, rabbit monoclonal, clone C70B1 (dilution: 1:1000)<br>ACTIN, Santa Cruz, sc-47778, mouse monoclonal, clone C4 (dilution: 1:2500)<br>BRD4, Bethyl, A301-985A100, rabbit polyclonal (dilution: 1:1000)<br>goat anti-rabbit IRDye 800CW, LI-COR, 925-32211 (dilution: 1:10,000)<br>goat anti-mouse IRDye 680CW, LI-COR, 925-68070 (dilution: 1:10,000)
Validation	Antibodies used in ChIP or HiChIP:<br>H3K27ac (Abcam, ab4729) has been validated for ChIP assays in human cells included in the ENCODE project (PMID: 22955616) as well as HiChIP assays in human cells (PMID: 28945252).<br>H3K27ac (Active Motif 39133) has been validated for ChIP assays in human cells included in the ENCODE project (PMID: 22955616) as well as HiChIP assays in human cells (PMID: 31784732).<br>BRD4 (Bethyl, A301-985A100) has been validated for ChIP assays in human cells based on published ChIP-seq results in T-ALL cell lines (PMID: 28673542).<br>SOX2 (R&D Systems, AF2018) has been validated for ChIP assays in human cells based on published ChIP-seq results in esophageal squamous cancer cell lines KYSE70, TT, and HCC95 (PMID: 24590290).<br>CTCF (Cell Signaling, 2899) has been validated for ChIP assays in human cells based on manufacture's ChIP-qPCR results in HeLa cells (https://www.cellsignal.com/products/primary-antibodies/ctcf-antibody/2899).<br>FOSL1 (Cell Signaling, 5281, clone D80B4) has been validated for ChIP assays in human cells based on previous literature in the head and neck squamous cancer cell line SCC1 (PMID: 33794365).<br>Cas9 (Diagenode, C15310258) has been validated for dCas9 ChIP assays in human cells based on the manufacture's ChIP-qPCR results in Jurkat and HEK293T cells expressing dCas9 (https://www.diagenode.com/en/p/crispr-cas9-polyclonal-antibody).<br><br>Antibodies used for Immunoblotting:<br>SOX2 (Cell Signaling, 3728, clone C70B1) has been validated for immunoblotting in the human cell line PC3 by previous research (PMID: 27196761). In this study, we validated the specificity of the antibody by using human cells with and without ectopic expression of SOX2 cDNA (Supplementary Figure 4b) or human cells with and without CRISPR-mediated SOX2 knockout (Supplementary Figure 4c).<br>ACTIN (Santa Cruz, sc-47778, clone C4) has been validated for immunoblotting in human cell lines such as HeLa, Jurkat, K562 etc . by the manufacture (https://www.scbt.com/p/beta-actin-antibody-c4).<br>BRD4 (Bethyl, A301-985A100) has been validated for immunoblotting in human cell lines including HEK293T, A549, HepG2 etc. by the manufacture (https://www.bethyl.com/product/A301-985A100/BRD4+Antibody).

Eukaryotic cell lines

Policy information about cell lines

Cell line source(s)	Squamous cancer cell lines KYSE140, KYSE70, LK2, NCI-H520, HSC4, TE1, TE10, SKMES1, and RERFLCAI were obtained from the Broad Institute Cancer Cell Line Encyclopedia (CCLE). The esophageal squamous cancer cell line TT was obtained from Japanese Collection of Research Bioresources (JCRB) Cell Bank.
Authentication	Cell line identities were verified by either SNP-array-based fingerprinting as previously described in the CCLE project or Short Tandem Repeats (STR) analysis.
Mycoplasma contamination	All cell lines were tested negative for mycoplasma using the Lonza MycoAlert Detection kit.
Commonly misidentified lines<br>(See ICLAC register)	No commonly misidentified cell lines were used in the study.

Animals and other organisms

Policy information about studies involving animals; ARRIVE guidelines recommended for reporting animal research

Laboratory animals	For xenograft experiments, we used 6-8 weeks old female nude mice (Nu/Nu) purchased from Jackson Laboratory. All the mice were housed in pathogen-free environment, with 12 hours of light and 12 hours of dark cycles, 18-21C degrees, and 40-60% humidity.
Wild animals	No wild animals were used in the study.
Field-collected samples	No field collected samples were used in the study.
Ethics oversight	All animal experiments were conducted in accordance with procedures approved by the institutional Animal Care and Use Committee at the Dana-Farber Cancer Institute, in compliance with NIH guidelines.

Note that full information on the approval of the study protocol must also be provided in the manuscript.

ChIP-seq

Data deposition

- [x] Confirm that both raw and final processed data have been deposited in a public database such as GEO.
- [x] Confirm that you have deposited or provided access to graph files (e.g. BED files) for the called peaks.

Data access links<br>*May remain private before publication.*	GEO (accession #GSE166234). URL: https://www.ncbi.nlm.nih.gov/geo/query/acc.cgi?acc=GSE166234
Files in database submission	GSM5066565 KYSE140_H3K27ac<br>GSM5066566 KYSE140_DMSO2hrs_H3K27ac_A<br>GSM5066567 KYSE140_DMSO2hrs_H3K27ac_B<br>GSM5066568 KYSE140_ARV2hrs_H3K27ac_A<br>GSM5066569 KYSE140_ARV2hrs_H3K27ac_B<br>GSM5066570 KYSE140_sg_NC1_BRD4<br>GSM5066571 KYSE140_sg_NC2_BRD4<br>GSM5066572 KYSE140_sg_e1_1_BRD4<br>GSM5066573 KYSE140_sg_e1_2_BRD4<br>GSM5066574 LK2_sg_NC1_BRD4<br>GSM5066575 LK2_sg_e1_1_BRD4<br>GSM5066576 H520_sg_NC1_BRD4<br>GSM5066577 H520_sg_e1_1_BRD4<br>GSM5066578 HSC4_sg_NC1_BRD4<br>GSM5066579 HSC4_sg_e1_1_BRD4<br>GSM5066580 KYSE140_DMSO2hrs_BRD4_A<br>GSM5066581 KYSE140_DMSO2hrs_BRD4_B<br>GSM5066582 KYSE140_ARV2hrs_BRD4_A<br>GSM5066583 KYSE140_ARV2hrs_BRD4_B<br>GSM5066584 KYSE140_SOX2<br>GSM5066585 KYSE140_CTCF
Genome browser session<br>(e.g. UCSC)	No longer applicable.

Methodology

Replicates	For comparing ChIP-seq signal between different conditions, one or two biological replicates were used. For profiling H3K27ac, SOX2, and CTCF binding in KYSE140 cells, one sample was used for each factor.
Sequencing depth	Please see detailed information of the deposited datasets in GEO (accession #GSE166234). URL: https://www.ncbi.nlm.nih.gov/geo/query/acc.cgi?acc=GSE166234
Antibodies	H3K27ac, Abcam, ab4729 (4ug/ChIP)<br>BRD4, Bethyl, A301-985A100 (4ug/ChIP)<br>SOX2, R&D Systems, AF2018 (4ug/ChIP)<br>CTCF, Cell Signaling, 2899 (10ul/ChIP)
Peak calling parameters	For narrowPeaks: macs2 callpeak -t bam_file -f BAMPE -n name -B --SPMR<br>For broadPeaks: macs2 callpeak -t bam_file -f BAMPE -n name -B --SPMR --broad
Data quality	We used q-value of 0.05 to select significant ChIP-seq peaks.
Software	Bowtie2 (2-2.2.9) for ChIP-seq and RNA-seq reads alignment<br>samtools (1.12) for sorting and indexing alignments<br>MACS2 (2.2.6) for ChIP-seq peak calling<br>bedtools (2.28.0) for ChIP-seq peaks comparison<br>Homer (v4.11) for super-enhancer identification<br>FIMO (5.0.5) for transcription factor motifs identification<br>deepTools (3.3.0) for ChIP-seq signal presentation
